# Palladium-Catalyzed Amination of Dichloroquinolines with Adamantane-Containing Amines

**DOI:** 10.3390/molecules18022096

**Published:** 2013-02-06

**Authors:** Anton S. Abel, Alexei D. Averin, Olga A. Maloshitskaya, Evgenii N. Savelyev, Boris S. Orlinson, Ivan A. Novakov, Irina P. Beletskaya

**Affiliations:** 1 Department of Chemistry, Lomonosov Moscow State University, Leninskie Gory, 1-3, Moscow 119991, Russia; E-Mails: antonabel@list.ru (A.S.A.); maloshitskaya@mail.ru (O.A.M.); beletska@org.chem.msu.ru (I.P.B.); 2 Volgograd State Technical University, 28 Lenina prosp., Volgograd 400131, Russia; E-Mails: phanchem@vstu.ru (E.N.S.; B.S.O.); rector@vstu.ru (I.A.N)

**Keywords:** amines, adamantane, Pd catalysis, amination, quinoline

## Abstract

Pd-catalyzed amination of isomeric 2,6-, 2,8-, 4,8- and 4,7-dichloroquinolines was studied using adamantane-containing amines in which substituents at the nitrogen atom differ in bulkiness. The selectivity of the amination of 2,6-dichloroquinoline was very low, substantially better results were obtained with 2,8-dichloroquinoline, and 4,8- and 4,7-dichloroquinolines provided the best yields of the amination products. Diamination of 4,8- and 4,7-dichloroquinolines was carried out with two amines which differ strongly in the bulkiness of the alkyl group. In the majority of cases BINAP ligand was successfully applied, however, it had to be replaced with DavePhos in certain reactions when using the most sterically hindered amine as well as for the diamination reactions.

## 1. Introduction

The pharmacological activity of adamantane derivatives is well documented and arises from various factors, the main being their ability to penetrate the lipid layers of membranes and to interact with hydrophobic sites of proteins due to the presence of a rigid lipophilic backbone. A special place among these compounds is occupied by the amines with adamantane-containing substituents which have already found medical applications, like adamantan-1-amine (amantadine) [1], (1-adamantylmethyl)amine hydrochloride (rimantadine) [2], 1,3-dimethyladamantan-5-amine (memantine) [3]. Biological studies of the adamantane derivatives bearing heterocyclic substituents, e.g., pyridine, pyrazole, benzimidazole, and other *N*-heterocycles are reported [4], and their psychotropic activity was especially addressed. However, there is scarce information about the quinolinyl derivatives of adamantaneamines, as only two publications [5,6] deal with 4-quinolinyl derivatives which demonstrated anti-malarial activity, and one describes the synthesis of 2-quinolinyl-substituted adamantane-1,3-diamine [7]. All these compounds were obtained using non-catalytic methods.

In general, the successful application of catalytic amination for the synthesis of aminoquinoline compounds from corresponding halogen derivatives depends strongly on the position of the halogen atom in the quinoline moiety. Pd-catalyzed amination reactions [8,9,10,11,12,13] are the most frequently used for this purpose. There are enough examples of the Pd-catalyzed amination of 2-chloroquinoline [14,15,16,17,18], 3-bromo- and 3-chloroquinolines [13,14,15,16,17,18,19,20,21,22,23], 6-bromoquinoline [24,25]. Less studied are the Pd-catalyzed aminations of 5-bromoquinoline [26,27], 8-chloro- and 8-bromoquinolines [14,28]. The Cu-catalyzed amination of 3-haloquinolines [29,30,31] and of 5-bromoquinoline [32] has also been reported, while there is almost no information about the application of the catalytic approaches for the synthesis of 4-aminoquinoline derivatives except for one work [33], although 4-amino- and especially 4-amino-7-chloroquinolines were shown to be potent anti-malarial agents [34,35,36].

Taking all these facts into consideration and in view of our interest in the Buchwald-Hartwig amination of heteroaryl halides [37,38,39,40], we decided to study the Pd-catalyzed amination reactions of isomeric dichloroquinolines with selected adamantane-containing amines, which differ in the steric hindrance at the amino group, and to determine the best conditions for the selective mono- and diamination of these hetaryl dichlorides.

## 2. Results and Discussion

**Scheme 1:**
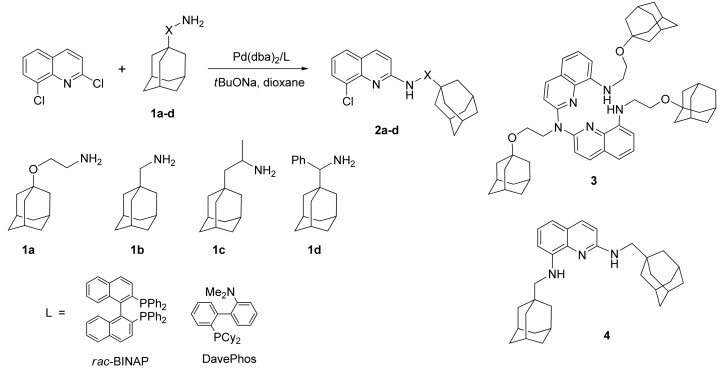
Pd-catalyzed amination of 2,8-dichloroquinoline.

We investigated the Pd-catalyzed amination of commercially available 2,6-, 2,8-, 4,8- and 4,7-dichloroquinolines which possess two chlorine atoms of different reactivity. The four adamantane-containing amines **1a**–**d** studied in the amination reactions have different substituents at the nitrogen atom. Catalytic reactions were carried out using Pd(dba)_2_ as a source of Pd(0), phosphine ligands BINAP (2,2'-bis(diphenylphosphino)-1,1'-binaphthalene) or DavePhos (2-dicyclohexylphosphino-2'-dimethylaminobiphenyl), and sodium *tert*-butoxide as a base. The reactions were conducted in boiling dioxane (c = 0.1 M), equimolar amounts of reagents were employed for the synthesis of monoaminosubstituted quinolines, 3–4 equiv. of amine were used to obtain diamino-substituted products. All four amines were studied in the monoamination reactions with each dichloroquinoline, and two amines, namely **1a** and **1d**, were employed in the diamination processes. Generally, the reactions were run for 6–8 h in the case of monoamination processes and 15 h in the case of diamination processes to achieve full consumption of the starting materials. The reaction products were isolated by column chromatography on silica gel. First, we carried out the reactions of 2,6-dichloroquinoline which was thought to be suitable for the synthesis of monoaminated products due to the presence of a reactive chlorine atom in position 2 and a much less reactive chlorine in position 6. However, all our attempts to obtain the monoamino or diamino derivative failed. The reactions with 1 or 4 equiv. of the amine **1a** catalyzed with 4 or 8 mol% of the catalyst, in the presence of BINAP or DavePhos, provided only complicated reaction mixtures which could not be separated by the chromatography. Such behavior of 2,6-dichloroquinoline may be explained by a high reactivity of the chlorine atom at position 2 which promotes the *N*-arylation and *N,N*-diarylation reactions as well as other substitution processes like alkoxylation with *t-*BuONa and homocoupling. Moreover, the substitution of the chlorine atom at position 2 markedly affects the substitution of the chlorine atom at position 6 due to a mesomeric effect. Thus we gave up attempts to work with 2,6-dichloroquinoline and turned to its isomer, 2,8-dichloroquinoline (Scheme 1).

This compound also possesses two chlorine atoms with distinctively different reactivity. The reaction with a less sterically hindered amine **1a** catalyzed by 4 mol% catalyst with BINAP as ligand provided 64% yield of the monoamination product **2a** in which a more reactive chlorine atom was substituted for the amino group (Table 1, entry 1). The attempt to obtain the diamination product by reacting 2,8-dichloroquinoline with 4 equiv. of the amine **1a** in the presence of the twofold amount of the same catalyst gave rise to an inseparable mixture (entry 2), and the diamination product could not be isolated as an individual product. The change of BINAP for the electron-donor DavePhos led to the formation of one main product **3**, which is the result of the combination of the diamination and *N,N*-diarylation processes (entry 3). It was isolated in 41% yield, and no desired diamino compound was obtained. The ^1^H-NMR spectrum of compound **3** is characterized by two distinctively different NCH_2_CH_2_O fragments. The first, bearing two quinolinyl substitutents at the nitrogen atom, possesses downfield-shifted CH_2_N protons (δ 4.58 ppm), while in the second fragment CH_2_N protons are observed at 3.4 ppm. In the ^13^C-NMR spectrum the corresponding carbon atoms are observed at 49.8 and 43.9 ppm, respectively. To compare, in the compound **2a** the protons of the CH_2_N group are observed at 3.7 ppm and corresponding carbon atom possesses the chemical shift 42.0 ppm. The downfield shift of both protons and carbon atoms in CH_2_NQuin_2_ fragment compared to CH_2_NHQuin is very characteristic [40] and verifies the structure of the compound **3**. Additional support is provided by a pronounced downfield shift of the H3, H3' protons of the quinolinyl moieties in compound **3** (7.47 ppm compared to 6.67 ppm in *N*-monoaryl derivative **2a**).

The reaction with one equivalent of a more sterically hindered amine **1b** unexpectedly gave not only the target monoamino derivative **2b**, but also some amount of the diamination product **4** (entry 4). This fact can be explained by the impossibility of *N,N*-diarylation in this case due to steric hindrances in the amine **1b**. Amine **1c**, in which the amino group bears a substituent with a tertiary carbon atom, is somewhat less active and provided 56% yield of the monoamination product **2c** (entry 5). It was necessary to increase the reaction time to 15 h to ensure full consumption of the starting materials. The reaction with the most sterically hindered amine **1d** could not be catalyzed with 4 mol% catalyst using BINAP (entry 6), but the application of DavePhos solved the problem, and the target compound **2d** was obtained in 42% yield (entry 8). It was found impossible to synthesize diaminosubstituted quinoline using the excess of amine and twofold amount of the catalyst either in the presence of BINAP or DavePhos ligands (entries 7 and 9). In all runs the reaction mixtures contained numerous compounds which could not be separated by the column chromatography. In many cases the formation of 2-*tert*-butoxy-substituted quinolines was noted, due to the non-catalytic substitution of the chlorine atom, and this process diminished the yields of the target amination products. The most upfield-shifted proton of the quinoline moiety in the compounds of type **2** (δ 6.6–6.7 ppm) possesses the largest coupling constant (^3^*J* = 8.8 Hz) with the most downfield-shifted proton (δ 7.7–7.8 ppm). This fact unambiguously supports the structure of these compounds with the amino group in position 2 of the quinoline system because ^3^*J*_H3H4_ is the largest coupling constant in quinolines and the difference in chemical shifts of these protons in 2-aminoquinolines is the biggest.

**Scheme 2:**
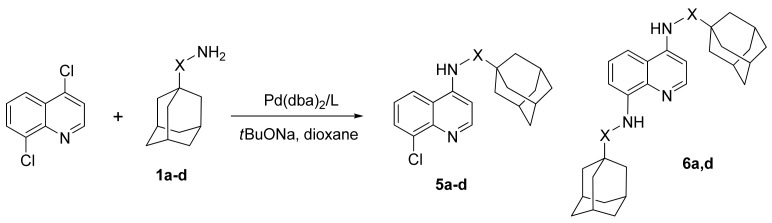
Pd-catalyzed amination of 4,8-dichloroquinoline.

If 2,8-dichloroquinoline was found to be quite capricious in the Pd-catalyzed amination, its isomer, 4,8-dichloroquinoline, proved to react in a smoother manner (Scheme 2). The reaction of the most active amine **1a** under the convenient catalytic conditions provided 77% yield of the product of the compound **5a** (Table 2, entry 1). It was impossible to obtain the 4,8-diaminosubstituted quinoline **6a** with 4 equiv. of this amine using BINAP as a ligand (entry 2), however, the application of DavePhos promoted this reaction (entry 3). The monoamination processes ran normally for all other amines **1b**–**d** and the yields of the 4-amino-8-chloroquinolines ranged from 67 to 84% (entries 4–6). These better results compared to the reactions with 2,8-dichloroquinoline can be attributed to a lower reactivity of the chlorine atom in the position 4 due to steric reasons and to the absence of the possible side reactions like *N,N*-diarylation. These results are in a good correspondence with our recent observations of the catalytic amination of 2- and 4-chloroquinolines [40]. The diamination of 4,8-dichloroquinoline with the most hindered amine **1d** is possible when using DavePhos ligand (entry 8), whereas in the presence of BINAP the reaction gave only the monoamination product **5d** almost in the same yield as it was with one equivalent of amine **1d** (entry 7). The most upfield-shifted proton of the quinoline moiety in the compounds of type **5** (δ 6.2–6.5 ppm) possesses the smallest coupling constant (^3^*J* = 5.4 Hz) with the most downfield-shifted proton (δ 8.4–8.6 ppm). This fact unambiguously supports the structure of these compounds with the amino group in the position 4 of the quinoline system because ^3^*J*_H2H3_ is the smallest coupling constant in quinolines and the difference in chemical shifts of these protons in 4-aminoquinolines is the biggest. The same observation is true for the compounds of type **7** (*vide infra*).

**Scheme 3:**
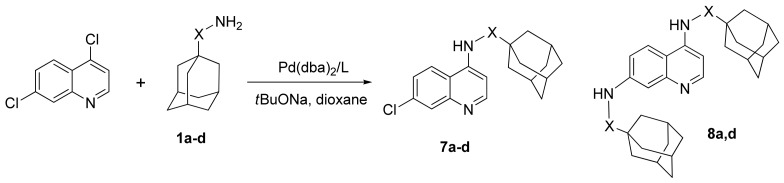
Pd-catalyzed amination of 4,7-dichloroquinoline.

As it has been already mentioned, the halogen atom in the position 7 of the quinoline ring is much less active than those in the positions 2 and 4, what should result in a better selectivity of the monoamination process. The reactions of 4,7-dichloroquinoline supported this idea (Scheme 3). The reaction with the amine **1a** afforded 52% yield of the 4-amino-7-chloroquinoline **7a** (Table 3, entry 1), while the diamination process was successful with either BINAP or DavePhos ligands (entries 2 and 3) providing almost equal amounts of the diaminated product **8a**. The reactions with the amines **1b**–**d** were successful as well, giving corresponding monoaminated products **7b**–**d** in 61–79% yields (entries 4–6). Diamination in the presence of Pd(dba)_2_/BINAP system ran normally, even with the most bulky amine **1d**, affording diamino derivative **8d** in 58% yield (entry 7). Diamino-substituted quinolines **6d** and **8d** are formed as pairs of diastereomers due to the presence of an asymmetric carbon atom in the structure of parent amine **1d**. Nevertheless, their ^1^H and ^13^C spectra show almost no splitting of the signals due to a long distance between the substituents bearing asymmetric carbon atom, their free rotation and the presence of a planar heteroaromatic spacer.

## 3. Experimental

### General

NMR spectra were registered using a Bruker Avance 400 spectrometer, MALDI-TOF spectra were obtained with a Bruker Autoflex II spectrometer using 1,8,9-trihydroxyanthracene as a matrix and PEGs as internal standards. Isomeric dichloroquinolines, BINAP and DavePhos ligands, sodium *tert*-butoxide were purchased from Aldrich and Acros and used without further purification. Amine **1a** was obtained according to a reported procedure [41], as was amine **1b** [42], while amines **1c**,**d** were obtained according to a method described in ref. [43]. Pd(dba)_2_ was synthesized from PdCl_2_ according to a known procedure [44]. Dioxane was distilled over NaOH followed by the distillation over sodium under argon. Acetonitrile, dichloromethane and methanol were used freshly distilled.

### Palladium-Catalyzed Amination of Dichloroquinolines—General Method

A two-neck flask equipped with a condenser and a magnetic stirrer, flushed with dry argon, was charged with corresponding dichloroquinoline (50 mg, 0.25 mmol), Pd(dba)_2_ (6–12 mg, 4–8 mol%), BINAP or DavePhos ligand (4.5–9 mol%), and absolute dioxane (2 mL). The mixture was stirred for 2–3 min, then corresponding amine **1a**–**d** (0.25 or 0.75–1 mmol) and *t*BuONa (0.375 mmol or 0.75 mmol) were added, and the reaction mixture was refluxed for 6–15 h. After cooling it down to ambient temperature the reaction mixture was diluted with CH_2_Cl_2_, the solution filtered and evaporated *in vacuo*, and the residue was chromatographed on silica gel using a sequence of eluents: petrol ether, petrol ether–CH_2_Cl_2_ 2:1–1:1, CH_2_Cl_2_, CH_2_Cl_2_/MeOH 200:1–3:1.

*N-[2-(1-Adamantyloxy)ethyl]-8-chloroquinolin-2-amine* (**2a**). Obtained from 2,8-dichloroquinoline (50 mg), amine **1a** (49 mg) in the presence of Pd(dba)_2_ (6 mg), BINAP (7 mg) and *t-*BuONa (36 mg). Eluent CH_2_Cl_2_/MeOH 100:1. Yield 57 mg (64%), yellowish viscous oil. ^1^H-NMR (CDCl_3_) δ 1.55–1.68 (m, 6H), 1.76 (br.s, 6H), 2.14 (br.s, 3H), 3.66–3.70 (m, 2H), 3.70–3.75 (m, 2H), 5.27 (br.s, 1H), 6.67 (d, *J* = 8.7 Hz, 1H), 7.08 (dd, *J_obs_* = 7.7, 7.7 Hz, 1H), 7.47 (d, *J* = 7.8 Hz, 1H), 7.62 (d, *J* = 7.5 Hz, 1H), 7.76 (d, *J* = 8.7 Hz, 1H). ^13^C-NMR (CDCl_3_) δ 30.5 (3C), 36.4 (3C), 41.6 (3C), 42.0 (1C), 58.7 (1C), 72.4 (1C), 112.6 (1C), 121.5 (1C), 124.5 (1C), 126.4 (1C), 129.5 (1C), 129.9 (1C), 137.3 (1C), 157.1 (1C), one quaternary carbon atom was not assigned due to line broadening. HRMS (MALDI-TOF): C_21_H_26_ClN_2_O (M+H)^+^ calcd.; 357.1734 observed; 357.1769.

*N^2^,N^8^-bis[2-(1-Adamantyloxy)ethyl]-N^2^-(8-{[2-(1-adamantyloxy)ethyl]amino}quinolin-2-yl)quinoline-2,8-diamine* (**3**). Obtained from 2,8-dichloroquinoline (50 mg), amine **1a** (195 mg) in the presence of Pd(dba)_2_ (12 mg), DavePhos (9 mg) and *t-*BuONa (60 mg). Eluent CH_2_Cl_2_/MeOH 200:1–100:1. Yield 57 mg (64%), yellowish viscous oil. ^1^H-NMR (CDCl_3_) δ 1.47–1.78 (m, 36H), 2.06 (br.s, 6H), 2.14 (br.s, 3H), 3.38–3.44 (m, 4H), 3.69 (t, *J* = 5.9 Hz, 4H), 3.84 (t, *J* = 6.0 Hz, 2H), 4.58 (t, *J* = 6.0 Hz, 2H), 6.09 (br.s, 2H), 6.67 (d, *J* = 7.6 Hz, 1H), 6.96 (d, *J* = 7.3 Hz, 1H), 7.21 (dd, *J_obs_* = 7.8, 7.8 Hz, 1H), 7.47 (d, *J* = 9.0 Hz, 1H), 7.87 (d, *J* = 9.0 Hz, 1H). ^13^C-NMR (CDCl_3_) δ 30.4 (9C), 36.4 (9C), 41.5 (9C), 43.9 (2C), 49.8 (1C), 58.0 (1C), 58.5 (2C), 72.2 (2C), 72.3 (1C), 105.4 (1C), 113.8 (1C), 116.4 (1C), 125.0 (1C), 125.5 (1C), 136.4 (1C), 137.0 (1C), 143.8 (1C), 153.9 (1C). HRMS (MALDI-TOF): C_54_H_70_N_5_O_3_ (M+H)^+^ calcd.; 836.5479 observed; 836.5422.

*N-(1-Adamantylmethyl)-8-chloroquinolin-2-amine* (**2b**). Obtained from 2,8-dichloroquinoline (50 mg), amine **1b** (41 mg) in the presence of Pd(dba)_2_ (6 mg), BINAP (7 mg) and *t-*BuONa (36 mg). Eluent petroleum ether–CH_2_Cl_2_ 1:1. Yield 34 mg (42%), yellowish viscous oil. ^1^H-NMR (CDCl_3_) δ 1.58–1.62 (m, 6H), 1.62–1.75 (m, 6H), 1.99 (br.s, 3H), 3.23 (d, *J* = 5.6 Hz, 2H), 5.03 (br.s, 1H), 6.70 (d, *J* = 8.8 Hz, 1H), 7.06 (dd, *J_obs_* = 7.7, 7.7 Hz, 1H), 7.46 (d, *J* = 8.0 Hz, 1H), 7.62 (d, *J* = 7.5 Hz, 1H), 7.78 (d, *J* = 8.8 Hz, 1H). ^13^C-NMR (CDCl_3_) δ 28.3 (3C), 34.2 (1C), 37.0 (3C), 40.5 (3C), 53.4 (1C), 111.5 (1C), 121.3 (1C), 124.4 (1C), 126.3 (1C), 129.6 (2C), 137.5 (1C), 143.5 (1C), 158.0 (1C). HRMS (MALDI-TOF): C_20_H_24_ClN_2_ (M+H)^+^ calcd.; 327.1628 observed; 327.1602.

*N,N'-bis(1-adamantylmethyl)quinoline-2,8-diamine* (**4**). Obtained as the second product in the synthesis of compound **2b**. Eluent petroleum ether–CH_2_Cl_2_ 1:1. Yield 16 mg (26%), yellowish viscous oil. ^1^H-NMR (CDCl_3_) δ 1.59–1.79 (m, 24H), 1.98 (br.s, 3H), 2.03 (br.s, 3H), 2.95 (d, *J* = 5.3 Hz, 2H), 3.28 (d, *J* = 6.4 Hz, 2H), 4.66 (br.s, 1H), 5.97 (br.s, 1H), 6.54–6.61 (m, 2H), 6.83 (d, *J* = 8.0 Hz, 1H), 7.05 (dd, *J_obs_* = 7.5, 7.5 Hz, 1H), 7.69 (d, *J* = 8.6 Hz, 1H). ^13^C-NMR (CDCl_3_) δ 28.4 (3С), 28.5 (3С), 34.0 (1C), 34.7 (1C), 37.1 (3C), 37.2 (3C), 40.8 (3C), 40.9 (3C), 53.1 (1C), 55.9 (1C), 105.0 (1C), 111.4 (1C), 113.4 (1C), 121.3 (1C), 122.6 (1C), 137.5 (1C), 143.2 (1C), 144.3 (1C), 155.5 (1C). HRMS (MALDI-TOF): C_31_H_42_N_3_ (M+H)^+^ calcd.; 456.3379 observed; 456.3425.

*N-[2-(1-Adamantyl)-1-methylethyl]-8-chloroquinolin-2-amine* (**2c**). Obtained from 2,8-dichloroquinoline (50 mg), amine **1c** (49 mg) in the presence of Pd(dba)_2_ (6 mg), BINAP (7 mg) and *t-*BuONa (36 mg). Eluent petroluem ether–CH_2_Cl_2_ 2:1–1:1. Yield 52 mg (56%), yellowish viscous oil. ^1^H-NMR (CDCl_3_) δ 1.27 (d, *J* = 6.4 Hz, 3H), 1.30 (dd, *J* = 14.4, 4.2 Hz, 1H), 1.39 (dd, *J* = 14.4, 7.3 Hz, 1H), 1.54–1.69 (m, 12H), 1.92 (br.s, 3H), 4.33 (br.s, 1H), 4.71 (br.s, 1H), 6.62 (d, *J* = 8.8 Hz, 1H), 7.06 (dd, *J_obs_* = 7.7, 7.7 Hz, 1H), 7.45 (dd, *J* = 7.8, 1.0 Hz, 1H), 7.62 (dd, *J* = 7.5, 1.0 Hz, 1H), 7.77 (d, *J* = 8.8 Hz, 1H). ^13^C-NMR (CDCl_3_) δ 23.7 (1C), 28.6 (3C), 32.5 (1C), 37.0 (3C), 42.9 (4C), 52.8 (1C), 111.7 (1C), 121.2 (1C), 124.3 (1C), 126.3 (1C), 129.5 (1C), 129.9 (1C), 137.4 (1C), 156.2 (1C), one quaternary carbon atom was not assigned due to line broadening. HRMS (MALDI-TOF): C_22_H_28_ClN_2_ (M+H)^+^ calcd.; 355.1941 observed; 355.1917.

*N-[1-Adamantyl(phenyl)methyl]-8-chloroquinolin-2-amine* (**2d**). Obtained from 2,8-dichloroquinoline (50 mg), amine **1d** (60 mg) in the presence of Pd(dba)_2_ (6 mg), DavePhos (5 mg) and *t-*BuONa (30 mg). Eluent petroleum ether–CH_2_Cl_2_ 2:1. Yield 42 mg (42%), yellowish viscous oil. ^1^H-NMR (CDCl_3_) δ 1.52–1.62 (m, 6H), 1.63–1.72 (m, 3H), 1.72–1.80 (m, 3H), 2.00 (br.s, 3H), 4.52 (br.s, 1H), 5.77 (br.s, 1H), 6.57 (d, *J* = 8.8 Hz, 1H), 7.03 (dd, *J_obs_* = 7.7, 7.7 Hz, 1H), 7.16–7.33 (m, 5H), 7.40 (d, *J* = 7.3 Hz, 1H), 7.59 (d, *J* = 7.3 Hz, 1H), 7.68 (d, *J* = 8.8 Hz, 1H). ^13^C-NMR (CDCl_3_) δ 28.4 (3C), 36.5 (1C), 36.8 (3C), 39.2 (3C), 66.0 (1C), 110.9 (1C), 121.4 (1C), 124.5 (1C), 126.3 (1C), 126.9 (1C), 127.6 (2C), 128.8 (2C), 129.2 (1C), 129.5 (1C), 137.6 (1C), 139.8 (1C), 144.3 (1C), 154.4 (1C). HRMS (MALDI-TOF): C_26_H_28_ClN_2_ (M+H)^+^ calcd.; 403.1941 observed; 403.1960.

*N-[2-(1-Adamantyloxy)ethyl]-8-chloroquinolin-4-amine* (**5a**). Obtained from 4,8-dichloroquinoline (50 mg), amine **1a** (49 mg) in the presence of Pd(dba)_2_ (6 mg), BINAP (7 mg) and *t-*BuONa (36 mg). Eluent CH_2_Cl_2_/MeOH 100:1–50:1. Yield 69 mg (77%), beige crystalline powder, m.p. 225–227 °C. ^1^H-NMR (CDCl_3_) δ 1.55–1.68 (m, 6H), 1.74–1.77 (m, 6H), 2.15 (br.s, 3H), 3.40 (q, *J* = 5.1 Hz, 2H), 3.73 (t, *J* = 5.2 Hz, 2H), 5.56 (br.s, 1H), 6.45 (d, *J* = 5.4 Hz, 1H), 7.31 (dd, *J_obs_* = 8.0, 8.0 Hz, 1H), 7.64 (d, *J* = 8.5 Hz, 1H), 7.74 (d, *J* = 7.5 Hz, 1H), 8.64 (d, *J* = 5.4 Hz, 1H). ^13^C-NMR (CDCl_3_) δ 30.4 (3C), 36.3 (3C), 41.6 (3C), 43.4 (1C), 57.5 (1C), 72.8 (1C), 99.7 (1C), 118.5 (1C), 120.2 (1C), 124.1 (1C), 129.2 (1C), 133.8 (1C), 144.8 (1C), 150.1 (1C), 151.4 (1C). HRMS (MALDI-TOF): C_21_H_26_ClN_2_O (M+H)^+^ calcd.; 357.1734 observed; 357.1698.

*N,N'-bis[2-(1-Adamantyloxy)ethyl]quinoline-4,8-diamine* (**6a**). Obtained from 4,8-dichloroquinoline (50 mg), amine **1a** (195 mg) in the presence of Pd(dba)_2_ (12 mg), DavePhos (9 mg) and *t-*BuONa (60 mg). Eluent CH_2_Cl_2_/MeOH 50:1. Yield 68 mg (52%), yellowish viscous oil. ^1^H-NMR (CDCl_3_) δ 1.55–1.75 (m, 12H), 1.77 (br.s, 12H), 2.13 (br.s, 3H), 2.16 (br.s, 3H), 3.37–3.46 (m, 4H), 3.73 (t, *J* = 5.8 Hz, 4H), 5.44 (br.s, 1H), 5.58 (br.s, 1H), 6.41 (d, *J* = 5.3 Hz, 1H), 6.67 (d, *J* = 7.7 Hz, 1H), 6.90 (d, *J* = 8.5 Hz, 1H), 7.27 (dd, *J_obs_* = 8.1, 8.1 Hz, 1H), 8.37 (d, *J* = 5.3 Hz, 1H). HRMS (MALDI-TOF): C_33_H_46_N_3_O_2_ (M+H)^+^ calcd.; 516.3590 observed; 516.3561.

*N-(1-Adamantylmethyl)-8-chloroquinolin-4-amine* (**5b**). Obtained from 4,8-dichloroquinoline (50 mg), amine **1b** (41 mg) in the presence of Pd(dba)_2_ (6 mg), BINAP (7 mg) and *t-*BuONa (36 mg). Eluent CH_2_Cl_2_/MeOH 100:1. Yield 56 mg (67%), beige crystalline powder, m.p. 210–212 °C. ^1^H-NMR (CDCl_3_) δ 1.58–1.61 (m, 6H), 1.61–1.76 (m, 6H), 2.00 (br.s, 3H), 2.69 (d, *J* = 5.7 Hz, 2H), 5.11 (t, *J* = 5.7 Hz, 1H), 6.47 (d, *J* = 5.4 Hz, 1H), 7.27 (dd, *J* = 8.6, 7.6 Hz, 1H), 7.63 (dd, *J* = 8.6, 1.1 Hz, 1H), 7.71 (dd, *J* = 7.6, 1.1 Hz, 1H), 8.60 (d, *J* = 5.4 Hz, 1H). ^13^C-NMR (CDCl_3_) δ 28.1 (3C), 34.0 (1C), 36.8 (3C), 40.6 (3C), 55.0 (1C), 99.4 (1C), 118.2 (1C), 119.9 (1C), 123.9 (1C), 129.1 (1C), 133.9 (1C), 144.8 (1C), 150.5 (1C), 151.4 (1C). HRMS (MALDI-TOF): C_20_H_24_ClN_2_ (M+H)^+^ calcd.; 327.1628 observed; 327.1654.

*N-[2-(1-adamantyl)-1-methylethyl]-8-chloroquinolin-4-amine* (**5c**). Obtained from 4,8-dichloroquinoline (50 mg), amine **1c** (49 mg) in the presence of Pd(dba)_2_ (6 mg), BINAP (7 mg) and *t-*BuONa (36 mg). Eluent CH_2_Cl_2_/MeOH 100:1–50:1. Yield 75 mg (84%), yellowish viscous oil. ^1^H-NMR (CDCl_3_) δ 1.25 (d, *J* = 6.4 Hz, 3H), 1.38 (dd, *J* = 14.6, 3.9 Hz, 1H), 1.45 (dd, *J* = 14.6, 7.6 Hz, 1H), 1.53 (br.s, 6H), 1.54–1.67 (m, 6H), 1.90 (br.s, 3H), 3.78–3.87 (m, 1H), 4.85 (d, *J* = 7.3 Hz, 1H), 6.48 (d, *J* = 5.5 Hz, 1H), 7.28 (dd, *J* = 8.6, 7.5 Hz, 1H), 7.60 (dd, *J* = 8.6, 1.1 Hz, 1H), 7.72 (dd, *J* = 7.5, 1.1 Hz, 1H), 8.64 (d, *J* = 5.5 Hz, 1H). ^13^C-NMR (CDCl_3_) δ 22.4 (1C), 28.5 (3C), 32.5 (1C), 36.8 (3C), 42.9 (3C), 44.2 (1C), 52.6 (1C), 99.2 (1C), 118.2 (1C), 120.0 (1C), 124.0 (1C), 129.2 (1C), 133.8 (1C), 145.0 (1C), 148.5 (1C), 151.4 (1C). HRMS (MALDI-TOF): C_22_H_28_ClN_2_ (M+H)^+^ calcd.; 355.1941 observed; 355.1978.

*N-[1-Adamantyl(phenyl)methyl]-8-chloroquinolin-4-amine* (**5d**). Obtained from 4,8-dichloroquinoline (50 mg), amine **1d** (60 mg) in the presence of Pd(dba)_2_ (6 mg), BINAP (7 mg) and *t-*BuONa (36 mg). Eluent CH_2_Cl_2_/MeOH 100:1. Yield 70 mg (70%), beige crystalline powder, m.p. 171–173 °C. ^1^H-NMR (CDCl_3_) δ 1.55–1.67 (m, 6H), 1.68–1.81 (m, 6H), 2.04 (br.s, 3H), 4.08 (d, *J* = 6.2 Hz, 1H), 5.77 (d, *J* = 6.2 Hz, 1H), 6.19 (d, *J* = 5.4 Hz, 1H), 7.20–7.32 (m, 5H), 7.38 (dd, *J_obs_* = 7.9, 7.9 Hz, 1H), 7.76 (d, *J* = 7.6 Hz, 1H), 7.79 (d, *J* = 8.3 Hz, 1H), 8.44 (d, *J* = 5.4 Hz, 1H). ^13^C-NMR (CDCl_3_) δ 28.2 (3C), 36.5 (1C), 36.7 (3C), 39.3 (3C), 67.2 (1C), 100.9 (1C), 117.7 (1C), 120.2 (1C), 124.2 (1C), 127.4 (1C), 127.9 (2C), 128.3 (2C), 129.1 (1C), 134.1 (1C), 137.9 (1C), 144.7 (1C), 149.0 (1C), 151.3 (1C). HRMS (MALDI-TOF): C_26_H_28_ClN_2_ (M+H)^+^ calcd.; 403.1941 observed; 403.1930.

*N,N'-bis[1-Adamantyl(phenyl)methyl]quinoline-4,8-diamine* (**6d**). Obtained from 4,8-dichloroquinoline (50 mg), amine **1d** (180 mg) in the presence of Pd(dba)_2_ (12 mg), DavePhos (9 mg) and *t-*BuONa (72 mg). Eluent petroleum ether–CH_2_Cl_2_ 1:1. Yield 75 mg (45%), beige crystalline powder, m.p. 175–177 °C. ^1^H-NMR (CDCl_3_) δ 1.50–1.83 (m, 24H), 1.95–2.04 (m, 6H), 4.00 (d, *J* = 6.6 Hz) + 4.02 (d, *J* = 6.7 Hz) (1H for two diastereomers), 4.06 (d, *J* = 5.0 Hz, 1H), 5.59 (d, *J* = 5.2 Hz, 1H), 6.11 (d, *J* = 5.2 Hz, 1H), 6.27 (d, *J* = 7.5 Hz, 1H), 6.91 (d, *J* = 8.3 Hz, 1H), 7.06–7.35 (m, 12H), 8.22 (d, *J* = 5.2 Hz, 1H). ^13^C-NMR (CDCl_3_) δ 28.3 (3C), 28.5 (3C), 36.6 (1C), 36.7 (1C), 36.8 (3C), 37.0 (3C), 39.3 (6C), 67.1 (1C), 67.8 (1C), 100.4 + 100.5 (1C for two diastereomers), 103.9 (1C), 105.0 (1C), 125.7 (1C), 126.6 (1C), 127.1 (1C), 127.4 (2C), 127.8 (2C), 128.4 (2C), 128.8 (2C), 138.1 (1C), 138.7 (1C), 140.3 (1C), 144.9 (1C), 145.0 (1C), 147.4 (1C), 148.7 (1C). HRMS (MALDI-TOF): C_43_H_50_N_3_ (M+H)^+^ calcd.; 608.4005 observed; 608.3969.

*N-[2-(1-adamantyloxy)ethyl]-7-chloroquinolin-4-amine* (**7a**). Obtained from 4,7-dichloroquinoline (50 mg), amine **1a** (49 mg) in the presence of Pd(dba)_2_ (6 mg), BINAP (7 mg) and *t-*BuONa (36 mg). Eluent CH_2_Cl_2_/MeOH 50:1–35:1. Yield 46 mg (52%), beige crystalline powder, m.p. 173–175 °C. ^1^H-NMR (CDCl_3_) δ 1.55–1.67 (m, 6H), 1.74–1.77 (m, 6H), 2.15 (br.s, 3H), 3.39 (q, *J* = 5.1 Hz, 2H), 3.72 (t, *J* = 5.2 Hz, 2H), 5.54 (br.s, 1H), 6.38 (d, *J* = 5.3 Hz, 1H), 7.35 (dd, *J* = 9.0, 2.2 Hz, 1H), 7.64 (d, *J* = 9.0 Hz, 1H), 7.93 (d, *J* = 2.2 Hz, 1H), 8.50 (d, *J* = 5.3 Hz, 1H). ^13^C-NMR (CDCl_3_) δ 30.4 (3C), 36.3 (3C), 41.6 (3C), 43.3 (1C), 57.6 (1C), 72.8 (1C), 99.2 (1C), 117.3 (1C), 121.0 (1C), 125.3 (1C), 128.6 (1C), 134.8 (1C), 149.0 (1C), 149.9 (1C), 151.9 (1C). HRMS (MALDI-TOF): C_21_H_26_ClN_2_O (M+H)^+^ calcd.; 357.1734 observed; 357.1715.

*N,N'-bis[2-(1-Adamantyloxy)ethyl]quinoline-4,7-diamine* (**8a**). Obtained from 4,7-dichloroquinoline (50 mg), amine **1a** (195 mg) in the presence of Pd(dba)_2_ (12 mg), DavePhos (9 mg) and *t-*BuONa (60 mg). Eluent CH_2_Cl_2_/MeOH 20:1–3:1. Yield 92 mg (71%), beige crystalline powder, m.p. 160–162 °C. ^1^H-NMR (CDCl_3_) δ 1.53–1.66 (m, 12H), 1.72 (br.s, 12H), 2.12 (br.s, 6H), 3.31 (q, *J* = 4.5 Hz, 2H), 3.42 (q, *J* = 5.1 Hz, 2H), 3.63 (t, *J* = 5.0 Hz, 2H), 3.70 (t, *J* = 5.4 Hz, 2H), 4.59 (br.s, 1H), 6.16 (br.s, 1H), 6.22 (d, *J* = 5.9 Hz, 1H), 6.79 (dd, *J* = 9.0, 1.9 Hz, 1H), 6.94 (d, *J* = 1.9 Hz, 1H), 7.62 (d, *J* = 9.0 Hz, 1H), 8.21 (d, *J* = 5.9 Hz, 1H). ^13^C-NMR (CDCl_3_) δ 30.4 (6C), 36.3 (6C), 41.5 (6C), 43.4 (1C), 43.9 (1C), 57.9 (2C), 72.4 (1C), 72.7 (1C), 99.7 (1C), 104.0 (1C), 110.6 (1C), 116.4 (1C), 121.0 (1C), 148.0 (1C), 148.1 (1C), 149.6 (1C), 151.2 (1C). HRMS (MALDI-TOF): C_33_H_46_N_3_O_2_ (M+H)^+^ calcd.; 516.3590 observed; 516.3634.

*N-(1-Adamantylmethyl)-7-chloroquinolin-4-amine* (**7b**). Obtained from 4,7-dichloroquinoline (50 mg), amine **1b** (41 mg) in the presence of Pd(dba)_2_ (6 mg), BINAP (7 mg) and *t-*BuONa (36 mg). Eluent CH_2_Cl_2_/MeOH 100:1–50:1. Yield 50 mg (61%), beige crystalline powder, m.p. 225–227 °C. ^1^H-NMR (CD_3_OD) δ 1.65 (br.s, 6H), 1.65–1.78 (m, 6H), 1.97 (br.s, 3H), 3.09 (s, 2H), 6.60 (d, *J* = 5.8 Hz, 1H), 7.39 (dd, *J* = 9.0, 2.1 Hz, 1H), 7.76 (d, *J* = 2.1 Hz, 1H), 8.13 (d, *J* = 9.0 Hz, 1H), 8.30 (d, *J* = 5.8 Hz, 1H), NH proton was not observed. ^13^C-NMR (CD_3_OD) δ 29.8 (3C), 36.7 (1C), 38.0 (3C), 41.7 (3C), 55.5 (1C), 100.0 (1C), 118.6 (1C), 124.2 (1C), 125.9 (1C), 127.5 (1C), 136.3 (1C), 149.7 (1C), 152.2 (1C), 153.8 (1C). HRMS (MALDI-TOF): C_20_H_24_ClN_2_ (M+H)^+^ calcd.; 327.1628 observed; 327.1649.

*N-[2-(1-Adamantyl)-1-methylethyl]-7-chloroquinolin-4-amine* (**7c**). Obtained from 4,7-dichloroquinoline (50 mg), amine **1c** (49 mg) in the presence of Pd(dba)_2_ (6 mg), BINAP (7 mg) and *t-*BuONa (36 mg). Eluent CH_2_Cl_2_/MeOH 50:1. Yield 73 mg (79%), beige crystalline powder, m.p. 207–209 °C. ^1^H-NMR (CDCl_3_) δ 1.26 (d, *J* = 6.2 Hz, 3H), 1.39 (dd, *J* = 14.8, 3.9 Hz, 1H), 1.46 (dd, *J* = 14.8, 7.6 Hz, 1H), 1.54 (br.s, 6H), 1.54–1.69 (m, 6H), 1.91 (br.s, 3H), 3.78–3.87 (m, 1H), 4.78 (d, *J* = 7.2 Hz, 1H), 6.42 (d, *J* = 5.5 Hz, 1H), 7.33 (dd, *J* = 9.1, 2.2 Hz, 1H), 7.60 (d, *J* = 9.1 Hz, 1H), 7.93 (d, *J* = 2.2 Hz, 1H), 8.51 (d, *J* = 5.5 Hz, 1H). ^13^C-NMR (CDCl_3_) δ 22.4 (1C), 28.5 (3C), 32.5 (1C), 36.8 (3C), 43.0 (3C), 44.1 (1C), 52.6 (1C), 98.8 (1C), 117.2 (1C), 120.7 (1C), 125.1 (1C), 128.8 (1C), 134.8 (1C), 148.2 (1C), 149.3 (1C), 152.0 (1C). HRMS (MALDI-TOF): C_22_H_28_ClN_2_ (M+H)^+^ calcd.; 355.1941 observed; 355.1904.

*N-[1-Adamantyl(phenyl)methyl]-7-chloroquinolin-4-amine* (**7d**). Obtained from 4,7-dichloroquinoline (50 mg), amine **1d** (60 mg) in the presence of Pd(dba)_2_ (6 mg), BINAP (7 mg) and *t-*BuONa (36 mg). Eluent CH_2_Cl_2_/MeOH 100:1. Yield 77 mg (77%), yellowish viscous oil. ^1^H-NMR (CDCl_3_) δ 1.54–1.66 (m, 6H), 1.68–1.80 (m, 6H), 2.03 (br.s, 3H), 4.07 (d, *J* = 6.2 Hz, 1H), 5.70 (d, *J* = 6.2 Hz, 1H), 6.13 (d, *J* = 5.3 Hz, 1H), 7.23–7.33 (m, 5H), 7.41 (dd, *J* = 8.8, 1.9 Hz, 1H), 7.79 (d, *J* = 8.8 Hz, 1H), 7.93 (d, *J* = 1.9 Hz, 1H), 8.31 (d, *J* = 5.3 Hz, 1H). ^13^C-NMR (CDCl_3_) δ 28.2 (3C), 36.5 (1C), 36.7 (3C), 39.3 (3C), 67.1 (1C), 100.4 (1C), 117.4 (1C), 120.4 (1C), 125.3 (1C), 127.4 (1C), 127.9 (2C), 128.7 (2C), 129.0 (1C), 134.6 (1C), 138.0 (1C), 148.7 (1C), 148.9 (1C), 151.9 (1C). HRMS (MALDI-TOF): C_26_H_28_ClN_2_ (M+H)^+^ calcd.; 403.1941 observed; 403.1958.

*N,N'-bis[1-Adamantyl(phenyl)methyl]quinoline-4,7-diamine* (**8d**). Obtained from 4,7-dichloroquinoline (50 mg), amine **1d** (180 mg) in the presence of Pd(dba)_2_ (12 mg), BINAP (14 mg) and *t-*BuONa (72 mg). Eluent CH_2_Cl_2_/MeOH 35:20:1. Yield 88 mg (58%), beige crystalline powder, m.p. 240–242 °C. ^1^H-NMR (CDCl_3_) δ 1.48–1.63 (m, 12H), 1.63–1.76 (m, 12H), 1.99 (br.s, 6H), 4.00–4.06 (2H), 4.74 (d, *J* = 6.7 Hz, 1H), 5.54 (d, *J* = 6.1 Hz), 5.85 (d, *J* = 5.4 Hz) + 5.86 (d, *J* = 5.4 Hz) (1H for two diastereomers), 6.81 (br.s, 1H), 6.85 (d, *J* = 9.0 Hz, 1H), 7.14–7.28 (m, 10H), 7.56 (d, *J* = 9.0 Hz, 1H), 8.07 (d, *J* = 5.4 Hz, 1H). ^13^C-NMR (CDCl_3_) δ 28.3 (3C), 28.4 (3C), 36.5 (2C), 36.8 (3C), 36.8 (3C), 39.2 (3C), 39.3 (3C), 66.9 (1C), 67.6 (1C), 97.1 + 97.8 (1C for two diastereomers), 107.1 (1C), 111.0 (1C), 115.7 (1C), 119.6 (1C), 126.9 (1C), 127.2 (1C), 127.7 (2C), 127.8 (2C), 128.3 (2C), 128.6 (2C), 138.5 (1C), 138.6 (1C), 139.5 (1C), 148.4 (1C), 149.1 (1C), 150.3 (1C). HRMS (MALDI-TOF): C_43_H_50_N_3_ (M+H)^+^ calcd.; 608.4005 observed; 608.3980.

## 4. Conclusions

To sum up, we have investigated the Pd-catalyzed amination reactions of isomeric chloroquinolines with several adamantane-containing amines and found out that 4,8- and 4,7-dichloroquinolines provided the best yields of the mono- and diamination reaction products due to a difference in the reactivity of the chlorine atoms. 2,8-Dichloroquinoline proved to be a more problematic substrate, especially in the case of the diamination process, and the application of 2,6-dichloroquinoline led only to complicated mixtures of unidentified products. The monoamination reactions were successfully performed using 1 equiv. of the amine and BINAP as a ligand, while diamination processes demanded the use of 3–4 equiv. of the amines and DavePhos ligand in the majority of cases. There was no pronounced dependence of the yields of the monoamination products on the bulkiness of the amine used, but the diamination processes were more successful for a less sterically hindered amine **1a** compared to the most hindered amine **1d**.

## Figures and Tables

**Table 1:** Pd-catalyzed amination of 2,8-dichloroquinoline.

Entry	Amine	Equiv. of amine	Ligand	Pd(dba)_2_/L, mol%	Product, yield
1	**1a**	1	BINAP	4/4.5	**2a**, 64%
2	**1a**	4	BINAP	8/9	inseparable mixture
3	**1a**	4	DavePhos	8/9	**3**, 41%
4	**1b**	1	BINAP	4/4.5	**2b**, 42%; **4**, 26%
5	**1c**	1	BINAP	4/4.5	**2c**, 56%
6	**1d**	1	BINAP	4/4.5	no amination
7	**1d**	3	BINAP	8/9	inseparable mixture
8	**1d**	1	DavePhos	4/4.5	**2d**, 42%
9	**1d**	3	DavePhos	8/9	inseparable mixture

**Table 2:** Pd-catalyzed amination of 4,8-dichloroquinoline.

Entry	Amine	Equiv. of amine	Ligand	Pd(dba)_2_/L, mol%	Product, yield
1	**1a**	1	BINAP	4/4.5	**5a**, 77%
2	**1a**	4	BINAP	8/9	inseparable mixture
3	**1a**	4	DavePhos	8/9	**6a**, 52% ^a^
4	**1b**	1	BINAP	4/4.5	**5b**, 67%
5	**1c**	1	BINAP	4/4.5	**5c**, 84%
6	**1d**	1	BINAP	4/4.5	**5d**, 70%
7	**1d**	3	BINAP	8/9	**5d**, 68%
8	**1d**	3	DavePhos	8/9	**6d**, 45%

^a^ Contains admixtures.

**Table 3:** Pd-catalyzed amination of 4,7-dichloroquinoline.

Entry	Amine	Equiv. of amine	Ligand	Pd(dba)_2_/L, mol%	Product, yield
1	**1a**	1	BINAP	4/4.5	**7a**, 52%
2	**1a**	4	BINAP	8/9	**8a**, 67%
3	**1a**	4	DavePhos	8/9	**8a**, 71%
4	**1b**	1	BINAP	4/4.5	**7b**, 61%
5	**1c**	1	BINAP	4/4.5	**7c**, 79%
6	**1d**	1	BINAP	4/4.5	**7d**, 77%
7	**1d**	3	BINAP	8/9	**8d**, 58%
